# Modern diagnostics for type B aortic dissection

**DOI:** 10.1007/s00772-015-0078-6

**Published:** 2015-10-05

**Authors:** T. Donati, J. Wilson, T. Kölbel, R.E. Clough

**Affiliations:** 1 Department of Vascular Surgery, Guy’s and St Thomas’ NHS Foundation Trust, London, UK; 2 Division of Imaging Sciences and Biomedical Engineering, King’s College London, St Thomas’ Hospital, Westminster Bridge Road, SE1 7EH London, UK; 3 Klinik und Poliklinik für Gefäßmedizin, Universitäres Herzzentrum Hamburg, Hamburg, Germany; 4 Guy’s, King’s and St Thomas’ Medical School, London, UK

**Keywords:** Computed tomography, Magnetic resonance imaging, Chest pain, Aortic dissection, Functional imaging, Computertomographie, Magnetresonanztomographie, Brustschmerz, Aortendissektion, Funktionelle Bildgebung

## Abstract

**Background:**

Undifferentiated chest pain is one of the most common complaints in the acute care setting. Type B aortic dissection is an important cause of chest pain and a complex clinical entity, which carries significant morbidity and mortality and requires accurate clinical and radiological evaluation.

**Methods:**

Imaging technologies have become an irreplaceable tool to establish the diagnosis of aortic dissection and to plan treatment strategies. Computed tomography is an important component in this process, replacing catheter-based angiography as the most commonly used preoperative and postoperative imaging modality for the thoracic aorta. The use of functional imaging methods, such as magnetic resonance imaging and echocardiography is evolving. These methods are able to provide the clinically relevant anatomical, hemodynamic and biomechanical information that is necessary for accurate diagnosis, risk stratification and patient selection for treatment.

**Conclusion:**

Advanced image acquisition equipment and expertise are increasingly available in a growing number of institutions and as a consequence, existing strategies for the management of type B dissection are rapidly evolving.

## Abstract (de)

**Hintergrund:**

Der akute Thoraxschmerz stellt eine der häufigsten Beschwerden in der medizinischen Akutversorgung dar. Typ-B-Aortendissektion ist eine wichtige Ursache von Thoraxschmerz und ein komplexes klinisches Krankheitsbild, das mit erheblicher Morbidität und Mortalität verbunden ist. Thoraxschmerz erfordert daher eine präzise klinische und radiologische Beurteilung sowohl im akuten als auch im chronischen Stadium.

**Methoden:**

Bildgebende Verfahren sind heute unersetzlicher Teil der Dissektionsdiagnose und der Planung von Behandlungsstrategien. Die Computertomographie (CT) ist eine wichtige Komponente in diesem Prozess und ersetzt digitale Subtraktionsangiographie als das häufigste prä- und postoperative Verfahren für die Bildgebung der Aorta. Funktionelle Modalitäten, wie die Magnetresonanztomographie (MRT) und die Echokardiographie, entwickeln sich weiter und sind in der Lage, die klinisch relevanten anatomischen, hämodynamischen und biomechanischen Informationen, die für eine genaue Diagnose, Risikostratifizierung und Auswahl der Behandlungsstrategie notwendig sind, zur Verfügung stellen.

**Schlussfolgerung:**

Die notwendige technische Ausrüstung und das Knowhow für moderne Bildgebung sind in einer wachsenden Zahl von Institutionen verfügbar, und die derzeitigen Strategien für die Behandlung von Typ-B-Dissektion entwickeln sich schnell.

## Introduction

Undifferentiated chest pain is one of the most common complaints in the acute care setting, accounting for over 5 million emergency department visits in the USA each year [1]. The cost to manage patients with non-specific acute chest pain is $10 billion in the USA annually [2]. The highest mortality of aortic dissection occurs in the first 48 h after symptom onset and therefore early diagnosis is important. One study reported that up to 39 % of aortic dissections have a diagnostic delay of at least 24 h [3].

Acute type B aortic dissection results in an intense inflammatory process and the development of complex hemodynamics that interrelate the true and false lumens [4, 5]. The disease is dynamic, particularly in this phase and changeable behavior of the intimal flap, extension of the dissection and expansion of the false lumen are possible. This can lead to contained or free rupture of the aorta, organ and/or limb malperfusion, early false lumen expansion, resistant hypertension and uncontrollable pain [6]. Dissections with these features are considered to be complicated and endovascular intervention is indicated. Dissections without these features are considered to be uncomplicated and are currently treated with best medical therapy [7]. The boundary between complicated and uncomplicated dissection is, however, blurred and if anatomically suitable, pre-emptive endovascular treatment has been advocated in uncomplicated dissections as a viable alternative to medical treatment alone [8]. There are, however, considerable risks associated with endovascular repair, such as stroke and paraplegia and careful consideration should be given before its use in all cases [9].

The growing availability and technical advances in modern imaging provide a way to develop an understanding of the complex mechanisms that govern aortic dissection [10]. Patient characteristics based on imaging data have been investigated to try to distinguish different subsets of patients. Morphological and false lumen characteristics including diameter, position, size and number of entry tears have been shown to serve as predictors of outcome, which may be used to identify high-risk patients [11]. An ideal imaging modality in the context of thoracic aortic disease should be able to evaluate the extent of the disease, the position and patency of side branches, identify high-risk features, allow procedure planning and postoperative evaluation. In many situations a combination of imaging modalities may provide the greatest benefit.

## Imaging modalities

Computed tomography (CT) angiography, magnetic resonance (MR) angiography and echocardiography are the techniques most commonly used in clinical practice for the assessment of aortic dissection (Table 1).

**Table 1:** Comparison of imaging techniques with respect to advantages and limitations (*CT* computed tomography, *MRI* magnetic resonance imaging, *TTE* transthoracic echocardiography, *TEE* transesophageal echocardiography)

Modality	Advantages	Limitations
CT	Widely available	Exposure to ionizing radiation
	Quick acquisition times	Need for iodinated contrast media
	Evaluation of entire aorta, its branches and surrounding organs	No functional/dynamic assessment of the heart and the aorta
	Allows evaluation of the iliac/femoral artery access	
MRI	High resolution images of the aorta and the aortic wall	Availability (especially in emergency setting)
	Does not require ionizing radiation or iodinated contrast media (ideal for surveillance)	Not for use in unstable patients
	Can provide functional information	Limited assessment of access (calcifications)
		Longer acquisition times
TTE	Portable and widely available	Poor/insufficient assessment of aorta distal to ascending aorta
	Quick assessment of cardiac function, ascending aorta and pericardium	Needs to be combined with another imaging modality for a thorough assessment of aortic dissection
		No role in surveillance/assessment of type B aortic dissection
TEE	High diagnostic accuracy in the thoracic aorta	Semi-invasive procedure, requiring sedation and is operator dependent
	Dynamic/functional assessment of heart and the aorta	Blind spot distal ascending aorta in the proximal arch
	Extremely valuable in the setting of endovascular procedures (patient monitoring/assessment of true and false lumen/positioning of the stent graft)	Limited/insufficient assessment of the entire aorta, visceral vessels, and access
		Needs to be combined with another imaging modality for thorough assessment of aortic dissection

### Computed tomography

Computed tomography (CT) angiography is widely available, has a rapid acquisition time, provides images with high spatial resolution and is the most commonly used imaging modality for anatomical evaluation of the thoracic aorta. State of the art scanners include multi-slice, multi-detector and multi-source technologies. This new technology results in a proportional decrease in the scanner time, which allows improved vascular opacification, with less contrast media, less aortic motion and fewer breathing artefacts [10]. The CT procedure is faster and more readily available than MRI and images of both the entire aorta and the aortic branch vessels can be acquired in a single scan. Non-contrast acquisitions provide valuable information about calcifications in the aortic wall that may be obscured after the contrast agent has been administered. Imaging without contrast should also be used initially to detect hemorrhage and hematomas [12]. The displacement of intimal calcification within the aortic lumen is a typical finding in aortic dissection.

Various CT protocols exist for evaluating patients with non-specific acute chest pain. Bolus tracking technology is the most commonly used technique to visualize the vessels. The length and density of the contrast agent bolus are important parameters in the optimization of this type of imaging, as both will affect the amount of contrast in the image [13]. The time at which the image data is acquired after the contrast agent has been delivered can also influence the contrast in the image and this forms the basis of arterial (early) and venous (late) phase imaging. Arterial phase images are used to assess the true lumen in the context of dissection and venous phase images are used to assess the false lumen. Patient comorbidities, for example heart failure or valve disease, can affect the quality of the images: in heart failure circulation times can be very slow, so that the images are triggered too early before the contrast agent has reached the diseased segment of the aorta and aortic valve incompetence can lead to unpredictable flow patterns, which in turn can result in erroneous early triggering when the region of interest is situated in the ascending aorta [10]. Triple phase protocols, consisting of a non-contrast-enhanced scan followed by both arterial and venous phase images, have been developed for aortic dissection. These scans help circumvent the difficulty in optimizing the time of data acquisition after the contrast bolus has been delivered. The triple rule out (TRO) protocol has been described as a CT examination for chest pain [14]. It is designed to differentiate between acute coronary syndrome, pulmonary embolism and acute aortic syndrome and is increasingly being performed in many institutions. These protocols are associated with higher doses of ionizing radiation compared with standard techniques [15].

**Fig. 1:**
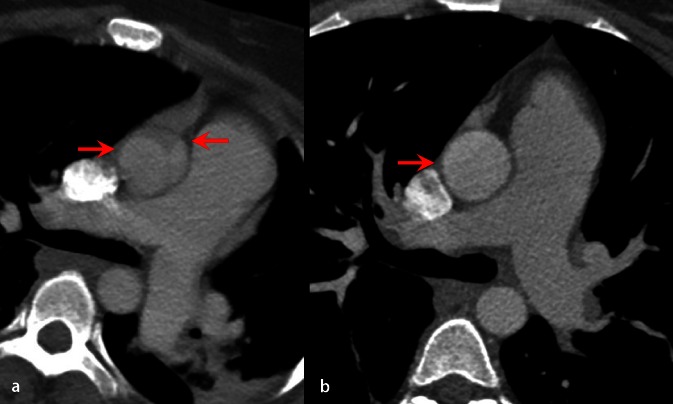
**a** Non-ECG-gated computed tomography image suggestive of type A dissection of the ascending aorta and **b** ECG-gated image 12 h later demonstrating the artefact was not a dissection and was caused by the overlying pulmonary artery

**Fig. 2:**
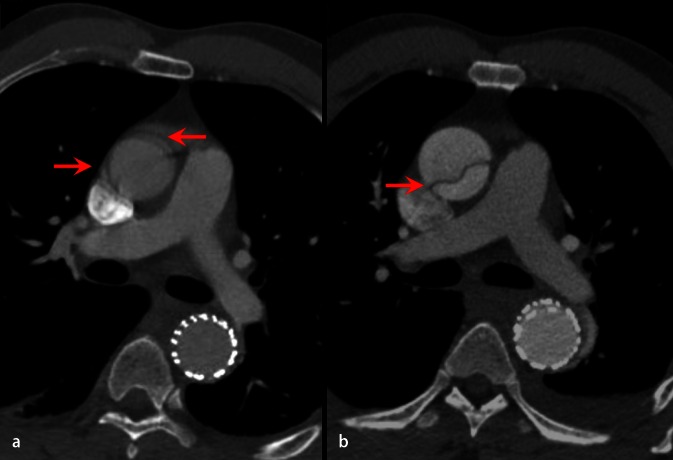
Computed tomography images acquired in the same imaging plane in a patient presenting with chest pain who had previously undergone endovascular repair of the descending thoracic aorta. Image **a** was acquired at presentation and image **b** 48 h later. The findings in image **a** (*arrows*) could represent artefacts or a new aortic dissection. At 48 h (image **b**) a clear dissection is seen in the ascending aorta (image **b** *arrow*). In the presence of a high clinical suspicion two imaging modalities should be used

The CT data can be acquired with reference to the electrocardiogram (ECG) signal to provide images of each phase of the cardiac cycle. This allows the acquisition of multiple three-dimensional (3D) images over time, so-called four-dimensional (4D) imaging. This, however, significantly increases the dose of ionizing radiation compared with conventional static scanning [15]. The very fast and complex movement of the aortic root during the cardiac cycle can result in motion artefacts in non-ECG-gated CT images. These artefacts can mimic an intraluminal flap and result in false positive and false negative diagnoses (Fig. 1). The use of ECG-gated CT allows acquisition of motion-free images during each phase of the cardiac cycle minimizing the risk of artefacts and it is an ideal imaging methods for evaluation of type A aortic dissection (Fig. 2). In addition, the coronary artery ostia and aortic valve function can also be assessed in the same scan [16]: ECG-gated CT can be used to assess branch vessel patency but the field of view is typically in the range of 30 cm in the head-foot direction so more than one scan may be required, resulting in very high doses of ionizing radiation.

**Fig. 3:**
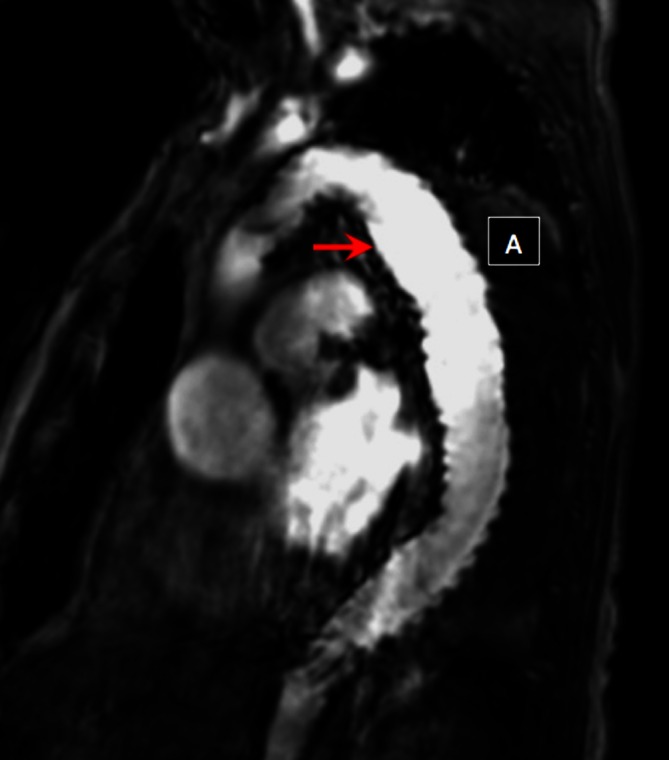
Magnetic resonance angiography of the thoracic aorta after endovascular repair demonstrating thrombosis of the false lumen (A). The stent graft is shown by the arrow.

Intraoperative CT utilizes cone beam reconstruction software and flat-panel detectors to generate CT-like images from rotational angiographic image acquisitions. This modality can be performed immediately in the operating theatre after completion of the endovascular procedure and offers the same benefits as CT imaging in terms of image quality and ability to reformat the image data in any plane [17]. The most commonly used modality to assess the thoracic aorta following endovascular repair is CT angiography. Usually only a contrast-enhanced scan is performed (omitting the non-enhanced scan), which helps to reduce the dose of ionizing radiation.

### Magnetic resonance imaging

Magnetic resonance imaging has some advantages over CT including superior soft tissue contrast, the absence of ionizing radiation and the ability to depict and quantify functional parameters [10]. Combining anatomical and functional information in a single acquisition means that MRI can provide a more comprehensive evaluation of thoracic aortic disease and is also an ideal modality for surveillance due to the absence of ionizing radiation, which is particularly important in this young patient population. The most commonly used MRI technique for both preprocedural and postprocedural imaging of the thoracic aorta is MR angiography (Fig. 3) [18]. Gadolinium-based contrast agents are used, which are less nephrotoxic compared to the iodinated agents used in CT imaging. In patients with very poor renal function these agents have been associated with the development of nephrogenic systemic fibrosis. In addition MRI has traditionally been associated with relatively long acquisition times and has therefore not been used in the acute setting.

First pass MRI is routinely used to measure false lumen thrombosis, where the image acquisition is timed according to the arrival of the contrast bolus in the proximal unaffected aorta and thrombosis is assumed to be present when there is no contrast agent in the false lumen. Recent studies have shown, however, that the flow rates in the false lumen are highly variable and often very slow [5, 19]. A recent study compared the amount of thrombus detected with standard clinical first pass MR and CT imaging with a new MRI technique using gadofosveset trisodium blood pool agent [13]. This study showed that first pass MRI and CT overestimated the amount of false lumen thrombosis by fivefold to sixfold. Imaging of the blood pool can also be acquired without the use of a contrast agent using an ECG-gated and respiratory-navigated balanced steady state free precession (SSFP) acquisition. The use of MR ECG-gating of SSFP imaging has been shown to provide better images of the proximal aorta compared to contrast-enhanced MR angiography because the latter technique does not use ECG gating.

Phase contrast (PC) sequences can be used to assess blood flow and velocity. They can also be used to acquire 3D velocity information (Vx, Vy, Vz) for each voxel, within a 3D volume over time (also known as 4D PC-MRI). This acquisition offers the potential to study aortic hemodynamics, flow patterns and derived vessel wall parameters, such as wall shear stress. This technique can help to identify entry tears between the true and false lumens and to stratify patients according to the risk of aneurysm formation [5, 20]. Dynamic imaging can also be useful to differentiate between dynamic and static branch vessel obstruction. Balanced cine-MRI slices can be used for this purpose, which have high temporal and spatial resolution and can be quickly acquired in any plane.

Black blood sequences can be used to assess the vessel wall, to evaluate hematoma and thrombus in the aortic wall and false lumen, respectively [12]. The 4D-TRAK is a contrast-enhanced MR angiography technique that can be used to acquire multiple time-resolved 3D volumes over time using image acceleration techniques [21]. It can be used to acquire several phases of contrast distribution, including but not limited to the arterial and venous phases. This technique can be used clinically to characterize flow-related phenomena, such as false lumen thrombus distribution and endoleaks: MRI is now possible after endovascular repair because the current generation of devices are largely made of non-ferrous materials, such as nitinol, which is MRI compatible. There are some emerging molecular MRI techniques to detect subclinical vascular disease where the amount of elastin in the aortic wall can be measured, which has the potential to identify patients with low levels of elastin at risk of aortic dilatation [22].

### Ultrasound imaging

Ultrasound techniques have relatively low spatial resolution compared with CT and MR imaging but they can provide functional information with high temporal resolution. A high clinical index of suspicion after a negative result from the first diagnostic investigation may warrant subsequent second or third imaging modalities. Transthoracic echocardiography (TTE) is useful in hemodynamically unstable patients because it is portable, rapid and the patient is still accessible during the acquisition of the imaging data [10]. This modality provides a good assessment of cardiac function, the aortic root and the proximal ascending aorta. Signs such as aortic dilatation, aortic regurgitation and pericardial effusion can be immediately recognized and suggest the possibility of aortic dissection. The TTE procedure is highly dependent on the operator for both acquisition and interpretation of the images, particularly in the acute setting [12]. The overlying lungs may make views of the descending thoracic aorta very limited. Focused cardiac ultrasound (FOCUS) compared with comprehensive ultrasound protocols can be useful for time-sensitive assessment and in suspected aortic dissection, aortic root size and valvular function and the presence of a dissection can be assessed [23].

**Fig. 4:**
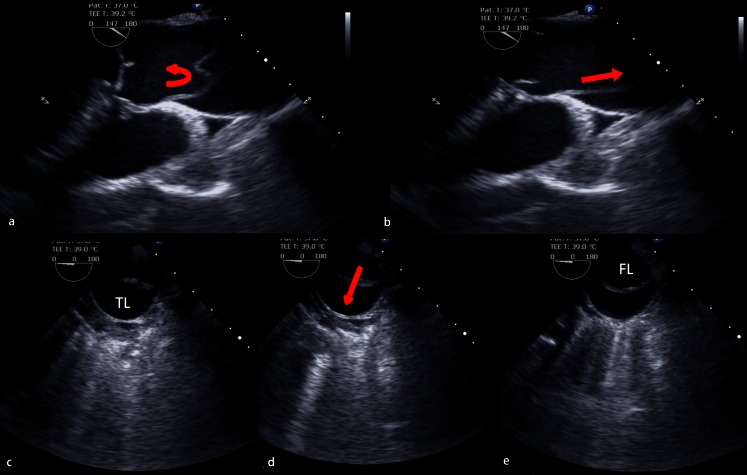
Transesophageal echocardiography (*TEE*) imaging demonstrating the infolding and unwinding (*arrows*) of the intimal flap at the level of the ascending aorta (**a**, **b**), and fluctuation of the intimal flap at the level of the descending aorta during the cardiac cycle (**c**, **d**, **e**) (*TL* true lumen, *FL* false lumen)

Transesophageal echocardiography (TEE) has a high accuracy for the diagnosis of aortic dissection and provides excellent anatomical and functional imaging of the descending thoracic aorta (Fig. 4; [10, 24]). In type B dissections it can be used to accurately recognized true and false lumens, proximal and distal reentry tears and can show the hemodynamic behavior of the intimal flap. Its main limitation is that no images are acquired below the diaphragm and because it is a semi-invasive procedure sedation is required. In the acute phase of a type B dissection its main role is probably for intraoperative assessment when endovascular treatment is required [25]. The images can be used to ensure the device is in the true lumen and positioned to cover the dominant entry tears. False positives can occur because of linear reverberation images, particularly in the ascending aorta. There are also blind areas, which limit assessment of the distal ascending aorta and proximal arch. The adjunct of color Doppler can facilitate identification of the true and false lumens.

Intravascular ultrasound (IVUS) involves the use of a probe that is advanced under fluoroscopic guidance. This technology is able to provide dynamic information regarding the true and false lumens and can detect false lumen thrombosis with higher sensitivity and specificity than TEE [26]. The IVUS procedure is especially useful to understand the precise mechanism of branch vessel compromise, evaluation of intramural hematoma and to identify the proximal entry tear [27]; however, IVUS is invasive and therefore its use in diagnosis and surveillance is limited.

### Positron emission tomography

Positron emission tomography (PET) is a functional imaging technique that works by measuring the distribution in the body of the tracer F-fluorodeoxyglucose (FDG). This is a glucose analog and has an increased uptake in cells with a high metabolic demand, such as inflammatory cells and cells in areas of acute injury. The use of FDG-PET scans, as well as higher resolution dual-modality scanning (PET/CT and PET/MRI), can therefore detect areas of inflammation and acute injury in large vessels and organs and has several applications that are being developed in aortic dissection. One such application is in the assessment of dissection chronicity, an important feature in deciding on treatment as well as predicting outcome [4, 28]. Acute dissections are associated with higher levels of FDG tracer uptake compared to chronic dissections. Other forms of imaging are unable to accurately assess this feature. The use of PET/CT scanning has also been suggested to be potentially valuable as a predictor of outcome in aortic dissection [29, 30]. Greater levels of FDG uptake in the dissected wall are associated with increased risk of poor outcome (e.g. rupture of the aorta or continued progression of the dissection); however, studies carried out so far have been of limited size and have not always shown consistent results and further research is needed in these areas to provide clearer guidance. The PET/CT procedure is also potentially useful in postoperative patients for the diagnosis of aortic stent graft infections, a possible complication of thoracic endovascular aortic repair (TEVAR).

## Conclusion

Imaging plays an important role in diagnosis, risk stratification and selection of the appropriate treatment in patients with thoracic aortic dissection. The decision of which diagnostic technique should be used depends on the availability of these techniques in the emergency situation and on local expertise. Future imaging techniques in aortic dissection should be used to detect early subclinical pathological changes and to identify patients who would benefit from early intervention.
